# The Impact of Device Settings, Use Patterns, and Flavorings on Carbonyl Emissions from Electronic Cigarettes

**DOI:** 10.3390/ijerph17165650

**Published:** 2020-08-05

**Authors:** Yeongkwon Son, Clifford Weisel, Olivia Wackowski, Stephan Schwander, Cristine Delnevo, Qingyu Meng

**Affiliations:** 1 Division of Atmospheric Sciences, Desert Research Institute, Reno, NV 89512, USA; 2 Department of Environmental and Occupational Health, School of Public Health, Rutgers University, Piscataway, NJ 08854, USA; weisel@eohsi.rutgers.edu (C.W.); schwansk@sph.rutgers.edu (S.S.); mengqi@sph.rutgers.edu (Q.M.); 3 Environmental and Occupational Health Sciences Institute, Rutgers University, Piscataway, NJ 08854, USA; 4 Center for Tobacco Studies, School of Public Health, Rutgers University, Piscataway, NJ 08854, USA; wackowol@sph.rutgers.edu (O.W.); delnevo@rutgers.edu (C.D.); 5 Cancer Prevention & Control Research Program, Cancer Institute of New Jersey, Rutgers University, New Brunswick, NJ 08901, USA; 6 Department of Urban-Global Public Health, School of Public Health, Rutgers University, Newark, NJ 07102, USA

**Keywords:** electronic cigarette, carbonyl, power, vaping topography, e-liquid, flavoring

## Abstract

Health impacts of electronic cigarette (e-cigarette) vaping are associated with the harmful chemicals emitted from e-cigarettes such as carbonyls. However, the levels of various carbonyl compounds under real-world vaping conditions have been understudied. This study evaluated the levels of carbonyl compounds (e.g., formaldehyde, acetaldehyde, glyoxal, and diacetyl, etc.) under various device settings (i.e., power output), vaping topographies, and e-liquid compositions (i.e., base liquid, flavor types). The results showed that e-vapor carbonyl levels were the highest under higher power outputs. The propylene glycol (PG)-based e-liquids generated higher formaldehyde and acetaldehyde than vegetable glycerin (VG)-based e-liquids. In addition, fruit flavored e-liquids (i.e., strawberry and dragon fruit) generated higher formaldehyde emissions than mint/menthol and creamy/sweet flavored e-liquids. While single-top coils formed 3.5-fold more formaldehyde per puff than conventional cigarette smoking, bottom coils generated 10–10,000 times less formaldehyde per puff. In general, increases in puff volume and longer puff durations generated significantly higher amounts of formaldehyde. While e-cigarettes emitted much lower levels of carbonyl compounds compared to conventional cigarettes, the presence of several toxic carbonyl compounds in e-cigarette vapor may still pose potential health risks for users without smoking history, including youth. Therefore, the public health administrations need to consider the vaping conditions which generated higher carbonyls, such as higher power output with PG e-liquid, when developing e-cigarette product standards.

## 1. Introduction

Carbonyl compounds are the most abundant toxic chemicals emitted from electronic cigarettes (e-cigarettes) [1,2,3,4]. Among the carbonyls found in e-cigarette emissions (e-vapor), two of the major carbonyls (i.e., formaldehyde and acetaldehyde) are known human carcinogens [5]. Glyoxal, which is known to cause allergic reactions, was also found in e-vapor [6]. Several flavoring carbonyls in e-liquids (e.g., vanillin, cinnamaldehyde) have shown increased cell toxicity [7,8]. It is also worth mentioning that 2,3-butanedione (diacetyl) and 2,3-pentanedione (acetylpropionyl) were found in e-vapors with ‘buttery’ flavored e-liquids [9] which are known to cause bronchiolitis obliterans (aka ‘popcorn lung’) [10]. However, factors affecting carbonyl emissions from e-cigarettes are still not fully understood. Most current studies report that higher e-cigarette power outputs significantly increase formaldehyde and acetaldehyde emissions [11,12,13,14,15,16,17]. However, there is a 1000-fold difference across the literature in formaldehyde emission rate data from e-cigarettes at comparable e-cigarette device power outputs [12,13,14], suggesting that carbonyl emissions are not dependent on e-cigarette power output alone and that other factors should be considered.

Among the potential other factors, flavoring agents used in e-liquids could be sources of potentially harmful carbonyl emissions. Thermal fragmentation of flavoring chemicals has been shown to form carbonyls under the burning temperatures of conventional cigarettes [18], but the contribution of flavoring chemicals to carbonyl emission has not been sufficiently studied for e-cigarettes [19,20]. In addition, some flavored e-liquids and e-vapors contain chemicals [e.g., diacetyl and acetylpropionyl] that can be toxic at sufficient levels [7,8,9]. However, levels of the potentially harmful chemicals in e-vapor resulting from flavoring additives were not fully accessed under e-cigarette vaping topography [9].

The contribution of e-liquid base materials to carbonyl emissions is also largely unknown. It has been shown that thermal degradation of vegetable glycerin (VG) and propylene glycol (PG), the main base materials used in e-liquids, generates various carbonyl compounds [15,21]. However, the thermal degradation of VG and PG has not been studied across a wide range of e-cigarette coil temperatures [14,16,22] even though coil temperature is an important determinant of e-cigarette carbonyl emissions [13].

E-cigarette vaping topography (i.e., puff volume and duration) could also potentially affect carbonyl formation. Vaping topography can affect carbonyl emissions by modifying e-cigarette heating coil temperatures [23]. However, most of the previous studies examining e-cigarette carbonyl emissions could not mimic actual e-cigarette user’s behavior [12,13,15,24,25]. Those studies generated e-vapors similar with the ‘Health Canada Intense (HCI) Regime’ (55 mL puff volume, 2 s puff duration, every 30 s) which was developed for conventional cigarette smokers and not e-cigarette vapors [26]. Therefore, real-world vaping topography should be used to study carbonyl emissions. In addition, most of the preceding studies only focused on formaldehyde and acetaldehyde emissions. However, other potentially harmful carbonyl compounds, such as acrolein and glyoxal, in e-vapor also need to be evaluated under real-world vaping conditions.

To address these knowledge gaps, this study evaluated the impacts of real-world vaping conditions (i.e., real-world e-cigarette heating power, vaping topography, and e-liquid components) on the emission of six potentially harmful carbonyls (i.e., glyoxal, formaldehyde, acetaldehyde, acrolein, diacetyl, and acetylpropionyl) and thirteen additional carbonyl species described below.

## 2. Materials and Methods

### 2.1. E-Vapor Generation Conditions and Materials

E-vapors were generated based on the protocols used in our previous studies [27,28,29]. In brief, vaping topographies of 23 current e-cigarette users measured in our lab. Vaping topographies (i.e., puff volume, duration, and interval) were measured using a CReSS Pocket device (Borgwaldt KC Incorporated, North Chesterfield, VA, USA) [30] with the Rutgers’ IRB approval (Pro20140000589). Demographic data of the study participants, observed vaping topographies, device settings, and e-liquid compositions from the users are summarized in Tables S1 and S2.

The wide ranges of experimental conditions were used reflecting real-world vaping conditions (Table 1). Throughout the experiments, the median values of the observed power output (6.4-watt), puff volume (90 mL), and puff duration (3.8 s) were used. The selected vaping topography was consistent with the median value of the reported e-cigarette vaping topographies, which was 91 mL puff volume (ranging from 51 to 133 mL) and 3.8 s puff duration (ranging from 2.65 to 4.3 s) [8,31,32,33,34,35,36,37]. Square-shaped vaping pattern was used (Figure S1) instead of bell-shaped topography which is used for cigarette smoking.

A variety of e-liquids were prepared to assess the impact of e-liquid compositions on carbonyl formation. Freshly prepared e-liquids in our lab were used in all experiments for quality control purposes because large variability in commercial products were reported in a previous study [38]. E-liquids used in our study were composed of vegetable glycerin (VG, USP grade, J.T. Baker, Phillipsburg, NJ, USA), propylene glycol (PG, USP grade, Sigma-Aldrich, St. Louis, MO, USA), (-)-nicotine (≥99.0%, Sigma-Aldrich), and flavoring agents (The Perfumer’s Apprentice, Scotts Valley, CA, USA). Three base materials, 100% VG, PG:VG mixture (*v/v* = 1:1), and 100% PG e-liquids, containing 12 mg/mL nicotine were tested to evaluate the impact of e-liquid base material on carbonyl formation. Then, eight flavored e-liquids (strawberry, dragon fruit, menthol, sweet cream, Bavarian, cinnamon, bubble gum, and graham cracker) were used to generate e-vapors to evaluate the impact of flavoring agents on carbonyl formation. These eight flavoring agents selected are the most frequently used ones in e-liquid recipes on the market [39]. The flavored e-liquids in the experiments were prepared by adding 10% of flavoring agents (1% for the cinnamon flavor) in VG by volume ratio. The chemical components of the flavoring agents were not fully disclosed but all of them were consisted of natural/artificial flavorings in PG as a main solvent (see more information in Supporting Information). All the e-liquids were prepared freshly before the day of experiments and sat overnight for stabilization. The e-liquid preparing procedures were consulted by local vape shop owners.

The device used in this study was a versatile refillable tank type e-cigarette which contained replaceable Nichrome heating coils (dual-bottom coils with 0.8 Ω resistance) and was obtained from an e-cigarette retailer (The Council of Vapor, Walnut Creek, CA, USA). Two types of battery boxes, an Apollo Valiant battery (Apolo E-cigarette, Concord, CA, USA) and a Sigelei-100W battery (Sigelei US, Pomona, CA, USA), were used to provide a wide range of power outputs from 3 to 80 watts. To test the impact of device power output settings and vaping topographies on carbonyl emission, the average and the 95% of the observed device power outputs (14.7 watts and 31.3 watts) and 95% of observed puff volume (170 mL) from the 23 subjects were used for e-vapor generation. The variable device power outputs were achieved by changing the battery voltage with fixed coil resistance (0.8 Ω).

### 2.2. Carbonyl Collection and Analysis

The sampling and analytical protocols for carbonyl measurements were developed based on the U.S. EPA compendium method OA-11A [40]. In brief, the sampling system was composed of a LXe1 smoking machine (Borgwaldt KC Incorporated, Hamburg, Germany), a sampling chamber, a 2,4-dinitrophenylhydrazine (DNPH) cartridge (Waters, Milford, MA, USA), and a vacuum pump (Figure S2). E-vapors were generated using the smoking machine under the experimental conditions described above and specified in Table S3. The generated e-vapors were directly introduced into the 2 L sampling chamber. The inlet of the DNPH cartridge was connected with the sampling chamber, and 30 puffs of e-vapor were collected using the DNPH cartridge with a sampling flow rate of 200 mL/min. After sampling, both ends of the DNPH cartridge were sealed, and the cartridge was stored in an aluminum zip lock bag at 4 °C until analysis.

The sampled DNPH cartridges were then eluted with 4 mL of high-performance liquid chromatography (HPLC) grade acetonitrile (ACN, Sigma-Aldrich), and 20 μL of the eluted DNPH-aldehyde derivatives were injected into the HPLC/UV system (a Perkin Elmer Series 200 HPLC and a Perkin Elmer 785a UV/vis detector, Perkin Elmer, MA, USA) which was equipped with a Waters Nova-Pak C18 column. The mobile phase was programmed as follows: after holding 100% of solvent A (H_2_O, HPLC grade, Sigma-Aldrich,/ACN/THF [tetrahydrofuran, Fisher Scientific, Hampton, NH, USA] = 6/3/1) for 4 min, the mobile phase was changed to 100% solvent B (ACN/H_2_O = 6:4) over 20 min, then 100% solvent B was held for 10 min. The mobile phase was set to a constant flow rate of 1 mL/min and the UV detector was set at an absorbance wavelength of 365 nm.

Calibration curves for the nineteen carbonyls were prepared using purchased DNPH-aldehyde analytical standards (ResTek, Bellefonte, PA, USA) and five carbonyl standards prepared in our lab (Table S3). For the preparation of these five carbonyl standards, glyoxal (40%) and vanillin (99%) were obtained from Sigma-Aldrich. Cinnamaldehyde (≥98), diacetyl (99%), and acetylpropionyl (97%) were purchased from Alfa Aesar (Haverhill, MA, USA). To prepare the standards, known amount of the five carbonyls were spiked into the DNPH cartridge and eluted with ACN. Limits of detection (LOD) and limits of quantification (LOQ) were three and ten times the standard deviations of the standard with the lowest concentration (*n* = 7).

### 2.3. Statistical Analysis

For all the experimental conditions, mean and standard deviations were estimated and presented. Two-tailed Student’s t-tests were conducted using R 3.4.3 (R Foundation, Vienna, Austria) to compare the mean values across different e-cigarette vaping conditions.

## 3. Results

### 3.1. The Impact of E-Liquid Base Materials and Power Outputs

**Figure 1:**
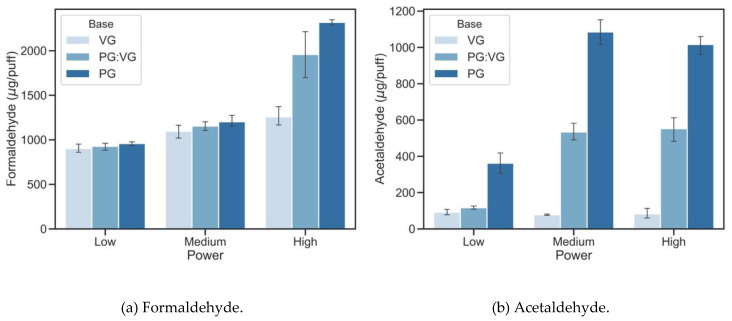
(**a**) Formaldehyde and (**b**) acetaldehyde levels (µg/puff) generated by combinations of base materials (100% VG, PG:VG 1:1 (*v/v*), and 100% PG) and device power outputs (6.4, 14.7, and 31.3 watt for low, medium, and high power, respectively). 90 mL puff volume, 3.8 s puff duration, and 24 s intervals was used as vaping topography, and 12 mg/mL nicotine was added into all e-liquids.

Figure 1 shows the impact of e-cigarette power output settings and variations in base material on the emission of formaldehyde and acetaldehyde, which are carcinogenic carbonyls found in e-vapor. Higher device power outputs increased formaldehyde emissions from all three base materials (Figure 1a). The amounts of formaldehyde generated at 31.3 watts were 39.3%, 111.0%, and 142.0% higher than the amounts of formaldehyde generated at 6.4 watts for VG, PG:VG, and PG based e-liquids, respectively. At 31.3 watts e-cigarette power output, PG:VG and PG based e-liquids generated 57.8% and 86.9% more formaldehyde than VG based e-liquids (*p* < 0.003).

PG-based e-liquids generated significantly higher amounts of acetaldehyde than VG-based e-liquids (Figure 1b). PG:VG mixture (*v*:*v* = 1:1) and PG based e-liquids generated 2.7 and 8.5 times more acetaldehyde than VG-based e-liquids at 6.4 watts power output condition (*p* < 0.001). The increase in e-cigarette power output from 6.4 watts to 31.1 watts did not increase acetaldehyde formation for VG e-liquid, but significantly increased for PG:VG and PG based e-liquids by a factor of 2.8–4.7 (*p* < 0.001). In addition to formaldehyde and acetaldehyde, higher power output also generated other harmful carbonyls (Table S4). We observed 240 ± 13.7 ng/puff of glyoxal generated from VG-based e-liquids at 31.3 watts. Compared with 6.4 watts conditions, 31.3 watts generated twice the amount of acrolein, *n*-butylaldehyde and isovaleraldehyde (*p* < 0.001). Interestingly, PG and PG:VG based e-liquids under higher power outputs emitted higher amount of *o*-tolualdehyde, while highest *n*-hexaldehyde were observed under medium power output (14.7 watt) for the all e-liquids.

### 3.2. The Impact of E-Liquid Base Materials and Power Outputs

**Figure 2:**
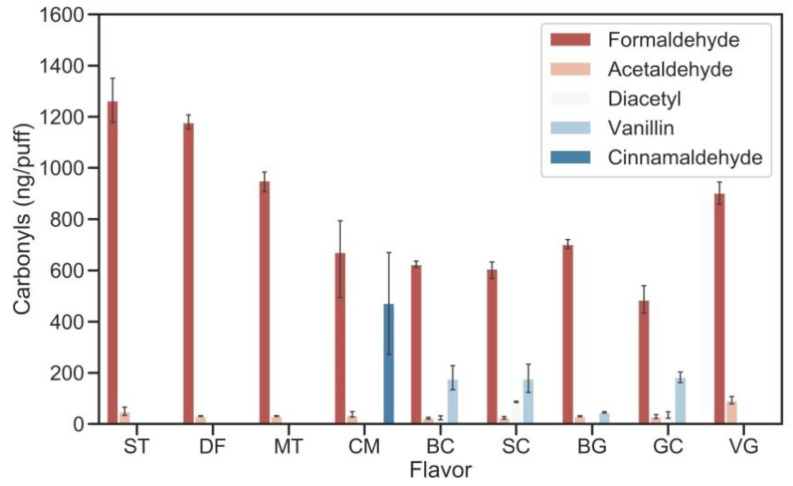
Carbonyl levels (ng/puff) generated by different flavored e-liquids (ST: strawberry, DF: dragon fruit, MT: menthol, CM: cinnamon, BC: Bavarian cream, SC: sweet cream, BG: bubble gum, and GC: graham cracker, 10% by volume, 1% for cinnamon flavor in VG-base) and non-flavored VG e-liquid (VG). Other vaping parameters are 6.4 watts power output, 90 mL puff volume, 3.8 s puff duration, and 24 s puff interval.

Carbonyl compounds generated from flavored e-liquids are shown in Figure 2 and Table S5. The fruit-flavored e-liquids (i.e., strawberry and dragon fruit) generated 1.7–2.6 times higher amounts of formaldehyde than spicy (cinnamon), and creamy/sweet (Bavarian cream, sweet cream, bubble gum, and graham cracker) flavored e-liquids.

The fruit and menthol flavored e-liquids generated 5–40% more formaldehyde than non-flavored VG e-liquid, while other flavored e-liquids generated less formaldehyde. In addition, acetaldehyde levels generated from the flavored e-liquids were below or similar to the quantification limit. Acrolein was generated at levels of approximately 20–30 ng/puff for 5 of the flavored e-liquids and more than 4 times of that level for Graham cracker flavor.

Diacetyl and acetylpropionyl are the ‘butter’ flavoring chemicals and are known to increase lung airway injury (aka ‘popcorn lung’) [41]. Three flavored e-liquid samples (i.e., Bavarian cream, sweet cream and graham cracker flavors) emitted 21.1–86.4 ng/puff of diacetyl, but acetylpropionyl was not detected in any sample. Four flavored e-liquids (i.e., Bavarian cream, sweet cream, bubble gum, and graham cracker flavors) emitted 45.2–184.4 ng/puff of vanillin, and cinnamaldehyde (473.1 ± 234.9 ng/puff) was detected only from the cinnamon flavored e-liquid.

### 3.3. The Impact of Vaping Topography

Variations in puff volumes and puff durations significantly changed carbonyl levels in e-vapors (Table 2). An increase in a puff volume from 35 mL to 90 mL led to 15.6% and 23.8% higher amounts of formaldehyde formation for 2 s and 3.8 s puffs, respectively (*p* < 0.016), while the mean formaldehyde emissions at 90mL and 170 mL puff volumes with same puff durations were not significantly different. Longer puff durations generated significantly higher amounts of formaldehyde. Acetaldehyde levels under the flow rates faster than 45 mL/s (i.e., 90 mL, 2 s puff and 170 mL, 2 s puff) were below limit of quantification. *o*-Tolualdehyde levels were higher at 2 s puff durations than 3.8 s puff durations at each puff volume, while higher *n*-hexaldehyde emissions were observed at 3.8 s than 2 s puff durations.

## 4. Discussion

This study adds new evidence on the levels of carbonyls emitted from e-cigarette. In this study, a variety of carbonyls were measured under wide ranges of e-cigarette use patterns: e-liquid compositions, power outputs, and vaping topography. The formation of carbonyls using various combinations of base materials and device power outputs were explored. Thermal decomposition of VG and PG forms carbonyls during e-cigarette vaping [21], and increased coil temperatures accelerate the decomposition rates of e-liquid base materials [42]. PG formed higher levels of carbonyl compounds (e.g., formaldehyde, acetaldehyde, butylaldehyde, tolualdehyde) during e-cigarette vaping than VG, probably because PG has a lower thermal decomposition temperature than VG. The thermal decomposition of PG starts as low as 127 °C [43], while VG requires at least 200 °C for its thermal decomposition reaction to begin [44]. Kosmider et al. [14] also reported that PG-containing e-liquids generated significantly higher amounts of formaldehyde and acetaldehyde than VG based e-liquids. Glyoxal, which was shown to cause allergic reaction [6], was observed only with VG-based e-liquid under high power output setting. It has been reported that thermal oxidation of VG leads to the formation of glyoxal [15].

**Figure 3:**
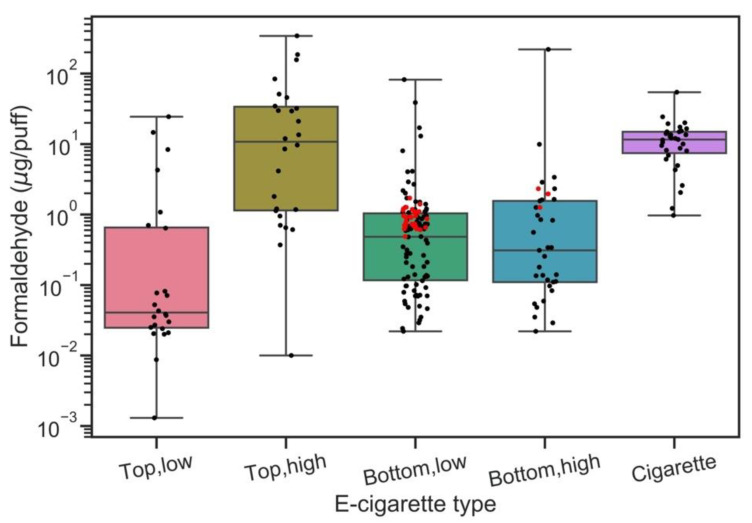
Formaldehyde levels (log-scale) from e-cigarettes with different coil structure (top- and bottom coil) and power outputs (low and high levels determined by lower and higher power outputs [5 watts for top coil and 18 watts for bottom coil] used in the literatures). Red dots indicate our results, and black dots are the results obtained from literatures [4,13,14,15,19,22,45,46,47,48,49]. Formaldehyde levels in cigarette smoke from literatures [50,51,52] are shown for comparison purpose.

Higher e-cigarette power outputs increased formaldehyde emissions for both top and bottom coils (Figure 3). The formaldehyde levels observed in this study were within interquartile ranges of literature values [4,13,15,45,46,47], but we observed wide varieties in reported formaldehyde levels. The reported e-vapor formaldehyde levels have been shown to range from 0.01 µg/puff to 342.2 µg/puff for top coil device and range from 0.02 µg/puff to 220.0 µg/puff for bottom coil e-cigarettes [4,13,14,15,19,22,45,46,47,48,49]. The wide range of e-cigarette carbonyl emission levels reported literatures might be a factor of the coil settings [13]. The top coils formed higher amounts of formaldehyde per puff (23.35 ± 59.68 µg/puff) with conventional cigarette smoking (12.32 ± 9.65 µg/puff) due to the limited e-liquid supply to the heating coil. A top coil is located on the top of the atomizer with long wicks dropping down into the e-liquid tank (Figure S3).

A long wick cannot supply enough e-liquid to the coil, and the limited e-liquid supply can easily dry up the heating coil, leading to a rapid coil temperature increase. The dramatic increase of coil temperature is known as ‘dry puff’ or ‘dry hit’, which results in significantly increased amounts of carbonyl formation [48]. In contrast, a bottom coil is located at the bottom of the atomizer, with a short wick contacting the e-liquid (Figure S3). Bottom-coils, commonly used in the current generations of e-cigarettes, generally provide consistent hits without ‘dry puffs’. Consequently, a bottom coil generated 10–10,000 times less formaldehyde per puff than conventional cigarettes due to stable e-liquid supply rates and coil temperatures. Gillman et al. [13] stated that e-cigarette devices with steady e-liquid supplies to the coil generated the lowest amounts of formaldehyde.

In addition to the device construction, variations in aldehyde emissions from flavored e-liquids might be affected by the differences in boil points, evaporation rates, and thermal decomposition rates [13]. But, the reasons for the differential carbonyl formation patterns across different flavoring agents are cannot be explored completely because flavor manufacturers usually do not disclose the ingredients in their products [53]. Based on partially revealed information from one vendor (The Perfumer’s Apprentice), the flavoring agents consist of PG, water, ethyl alcohol, and natural/artificial flavoring chemicals. Pyrolysis of flavoring chemicals was known to be the major source of carbonyls in e-vapor [19,20]. In addition, PG in flavoring agents might also contribute to aldehyde formation. However, current knowledges on carbonyl formation from flavored e-liquids are not fully understood. Further studies of thermal degradation of flavoring chemicals are warranted to better understand the contribution of flavoring agents to carbonyl formation.

Diacetyl concentrations observed in our samples are comparable to those reported in previous studies [9,37]. Even though many e-liquid manufacturers use acetoin as a safe alternative of diacetyl and acetylpropionyl, diacetyl could be found in e-vapor due to the use of natural flavors containing diacetyl and acetoin-to-diacetyl conversion during storage [10,54]. Based on the diacetyl concentrations we measured, assuming 200 puffs/day of e-cigarette vaping on average, daily diacetyl exposure levels of butter-like flavored e-cigarette users (4.22–17.28 g/day) were much lower than the reported threshold level. In a rodent in vivo study, diacetyl exposures (100 ppm) to 6 h per day for 12 weeks (equivalent to 6.82 mg/day for 12 weeks assuming 0.2 mL mean tidal volume and 250 breaths/minute) caused nasal injury and peribronchial lymphocytic inflammation [55]. However, considering uncertainties in animal-to-human extrapolation and extreme e-cigarette users (e.g., cloud chasers using sub-ohm e-cigarettes with intensive use, >1000 puffs/day), potential health impact of chronic low-level diacetyl exposures should be further accessed by the regulatory authorities. 

In addition to the diacetyl, other flavoring chemicals (e.g., vanillin and cinnamaldehyde, etc.) could promote adverse health outcomes. An in vitro study demonstrated that vanilla and cinnamon flavored e-liquids had three and ten-fold lower no-observable-adverse-effect-level (NOAEL) doses (0.1–0.01% dose) than VG only e-liquid (0.3% in culture media), respectively [7]. The cytotoxicity of cinnamaldehyde (IC_50_ = 0.037–0.04 mM) was approximately 100 times higher than that of vanillin (IC_50_ = 2.5–4 mM) [8]. The impact of flavoring chemicals on human health need to be further studied using real-world relevant doses, such as presented in this study, because the flavoring chemicals have been identified as one of the most concerning chemicals found in e-cigarette emissions [56,57].

Increased puff volumes with a fixed puff durations were shown not only to increase the amounts of e-vapor passing through the heating coil but also to decrease its temperature due to increased flow rates [23]. Puff volumes and puff durations determine the volume of air and its flow rate passing through the e-cigarette heating coils. The significant differences in carbonyl emissions measured between 35 mL and 90 mL puff volumes observed in our study might be due to the increased e-vapor masses. However, the carbonyl composition might also be affected by coil temperature changes. The formation of diverse carbonyl species under different air flow regimes might indicate that the changes in coil temperature could affect the thermal degradations of the e-liquid components.

Our study makes an important contribution to the literature by using vaping topographies based on real-world user vaping patterns. As noted earlier, the puff volumes used in most previous e-cigarette vapor emissions studies were based on regular cigarette smoking topographies and were usually much lower than that of e-cigarette users. The short puff durations used in previous studies (≤2 s) might be insufficient to heat up the heating coil to evaporate e-liquid [23] and thus may have underestimated potential exposures to toxic carbonyl emissions from e-cigarette vapor during real-world usage.

**Figure 4:**
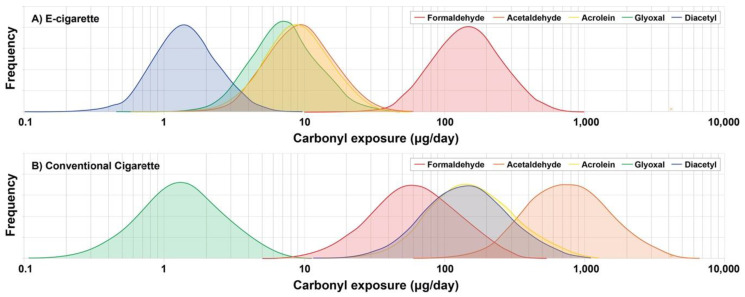
Estimated daily carbonyl exposures (µg/day) for (**A**) e-cigarette vapers and (**B**) conventional cigarette smokers.

Carbonyl exposure distributions for e-cigarette and conventional cigarette were estimated using the Monte Carlo method (Figure 4). Input parameters were the observed e-cigarette emission data in this study (Table 2 and Tables S4 and S5), reported cigarette smoke carbonyl levels (Table S6) [50], and daily e-cigarette and cigarette use patterns [31,58]. The distribution of carbonyl exposures associated with recent generation e-cigarettes with bottom coil setting were compared to the exposures from conventional cigarette smoking (Figure 4 and Table S7). Daily average acetaldehyde, diacetyl, and acrolein exposures from e-cigarette were approximately 100, 125 and 21 times lower than conventional cigarette, respectively, with little to no overlap of the exposure populations. However, e-cigarette users could be exposed to 2- and 4-fold higher formaldehyde and glyoxal in a day than cigarette smokers, respectively, and near complete overlap of the distributions. Given the daily exposure estimates, e-cigarette users should aware that e-cigarette might be less effective harm reduction product when they employ vaping conditions that resulted in high carbonyl formation (e.g., top-coil device, high power output, PG e-liquid, and large flavoring additives, etc.). In addition, e-cigarette vaping is still expected to pose potential health risks due to the non-threshold characteristics of carcinogenic carbonyls (i.e., formaldehyde and acetaldehyde) and should not be considered harmless. 

It is also worth mentioning that, as noticed above, e-cigarette users are prone to be exposed to more glyoxal than cigarette users. Glyoxal has been identified as an occupational allergen among health care workers who use glyoxal containing disinfectants [6]. An in vitro study showed that glyoxal was shown to deplete glutathione, increase the production of reactive oxygen species (ROS), and induced cell damage to isolated rat hepatocytes [59]. Higher device power outputs could increase glyoxal exposures and via the mechanisms listed above induce airway oxidative stress. Since there is no such study of health impacts of e-cigarette glyoxal exposure, it needs to be further evaluated for better harm reduction.

Moreover, carbonyl compounds in e-vapor were shown to form secondary harmful chemicals. Autoxidation of acetoin, which is a safer alternative of ‘butter’ flavoring chemicals (i.e., diacetyl and acetylpropionyl) could form diacetyl during e-liquid storage [54]. Further, acrylamide is neurotoxic and has potency to cause cancers in the reproductive and endocrine systems [60]. The reaction between acrolein, which is known to present in e-cigarette vapor, and amino acid or ammonia could form acrylamide [61], but the formation of acrylamide has not been studied in e-vapor. In addition to acetoin and acrolein, other precursor chemicals may present in e-vapors. Future research needs to access the formation of secondary air toxics induced by e-vapor.

Even though we thoughtfully identified large numbers of carbonyl compounds induced by various e-cigarette vaping conditions, this study still has several limitations. First, the DNPH cartridges were designed for the gas phase carbonyl sampling rather than particle phase carbonyls [40]. Carbonyl collection efficiencies for the e-vapors using the DNPH cartridges might be lower than the labeled efficiencies for the gas phase carbonyls because carbonyls in e-vapors are reported to be present in both gas and particle phases [15]. Second, our analytical method might have underestimated unsaturated aldehydes and ketones. Unsaturated carbonyls, such as acrolein, crotonaldehyde, and cinnamaldehyde, and DNPH adducts could further react with additional DNPH to form side products [62]. Further studies also need to test additional carbonyl sampling methods such as N-methyl-4-hydrazino-7-nitrobenzofurazan, 4-(2-aminooxyethyl)-morpholin-4-ium chloride or DNPH-hydroquinone methods.

## 5. Conclusions

E-vapor carbonyl levels vary under different vaping conditions and product materials. Higher carbonyl levels were found for PG e-liquids, higher power outputs, and top coil settings. PG-based e-liquids under 31.3-watt generated approximately 2.6, 11.2, and 200-fold higher formaldehyde, acetaldehyde, and acrolein than VG e-liquid under 6.4-watt. Despite the fact the FDA has announced a guideline that regulates e-cigarette flavorings for e-cigarette devices targeted to minors or marketed to target minors [63], our results are still useful because there is still huge flavored e-liquid market deemed to not target youth. It is worth mentioning that the flavored e-liquids that are not being sold after the guideline implementation (e.g., JUUL pods except for tobacco and menthol flavor) could always come back onto the market when the vendor meets the standards set out in the premarket authorization [63]. Our findings suggest that upper limits of e-cigarette power output need to be provided for different e-liquid compositions and coil types to minimize carbonyl emission. In addition, other potentially harmful carbonyls, diacetyl, vanillin, and cinnamaldehyde, were identified from the flavored e-liquids. Furthermore, flavored e-liquids changed the profile of carbonyl formation. However, the impact of flavoring chemicals could not be satisfactorily explored due to the limited availability of product content information. Future studies, therefore, will need to evaluate the impact of flavoring chemicals on the formation of carbonyls formation and the inhalation toxicity of flavoring chemicals by themselves.

## Figures and Tables

**Table 1:** E-vapor generation conditions used in this study ^1^.

Experiments	Factors	Settings	Other Settings
E-liquid flavor	Flavor	8 flavors ^2^	6.4 watt, 90 mL puff volume, 3.8 s puff duration, VG
	Flavoring level (%)	10% (*v/v*) ^3^	
E-liquid base	Base material	VG, PG:VG = 1:1 (*v/v*), PG	6.4 watt, 90 mL puff volume, 3.8 s puff duration
	Nicotine (mg/mL)	12	
Device setting	Device power (watt)	6.4, 14.7, 31.3	90 mL puff volume, 3.8 s puff duration, 12 mg/mL nicotine in VG
Vaping topography	Puff volume (mL)	35, 90, 170	6.4 W, 12 mg/mL nicotine in VG
	Puff duration (s)	2, 3.8	

^1^ 24 s puff intervals were used for all conditions; ^2^ Strawberry, Dragonfruit, Menthol, Cinnamon, Bubblegum, Bavarian cream, Sweet cream, and Graham cracker; ^3^ 1% for cinnamon flavor (*v/v*).

**Table 2:** Impact of vaping topography on carbonyl levels in e-vapor (mean ± standard deviation, ng/puff).

Carbonyl	Puff Volume and Duration ^†^					
	35 mL		90 mL		170 mL	
	2 s	3.8 s	2 s	3.8 s	2 s	3.8 s
	*n* = 3	*n* = 3	*n* = 3	*n* = 5	*n* = 3	*n* = 3
Glyoxal	ND ^††^	ND ^††^	ND ^††^	ND ^††^	ND ^††^	ND ^††^
Formaldehyde	683.0 ± 32.3	730.0 ± 53.8	790.0 ± 32.3	903.0 ± 56.2	747.0 ± 47.2	867.0 ± 32.7
Acetaldehyde	41.0 ± 9.35	39.9 ± 4.35	<LOQ ^††††^	91.7 ± 18.1	<LOQ ^††††^	47.1 ± 1.14
Acetone	<LOQ ^††††^	<LOD ^†††^	<LOQ ^††††^	<LOD ^†††^	<LOQ ^††††^	<LOD ^†††^
Acrolein	17.6 ± 1.32	32.0 ± 1.81	32.7 ± 1.27	<LOQ	28.9 ± 0.52	38.1 ± 1.52
Propionaldehyde	ND ^††^	ND ^††^	ND ^††^	ND ^††^	ND ^††^	ND ^††^
Crotonaldehyde	56.4 ± 3.82	34.9 ± 28.9	40.8 ± 0.56	29.8 ± 6.02	42.4 ± 0.67	ND ^††^
*n*-Butylaldehyde	ND ^††^	ND ^††^	ND ^††^	ND ^††^	ND ^††^	37.9 ± 1.80
Benzaldehyde	45.8 ± 1.41	31.4 ± 4.14	32.5 ± 0.46	23.1 ± 12.4	30.3 ± 2.45	29.5 ± 2.47
Isovaleraldehyde	ND ^††^	23.3 ± 5.56	33.4 ± 0.57	ND ^††^	ND ^††^	34.0 ± 1.55
*n*-Valeraldehyde	32.9 ± 12.8	ND ^††^	30.7 ± 2.18	81.1 ± 19.4	29.4 ± 0.92	ND ^††^
*o*-Tolualdehyde	134.1 ± 4.2	95.3 ± 17.9	196.0 ± 4.38	ND ^††^	186.0 ± 1.11	114. ± 4.19
*p*-Tolualdehyde	17.1 ± 0.26	21.6 ± 3.22	17.1 ± 0.18	18.1 ± 1.63	17.8 ± 0.15	20.4 ± 1.89
*n*-Hexaldehyde	ND ^††^	128.0 ± 8.06	ND ^††^	248.0 ± 65.1	ND ^††^	413.0 ± 4.65
Dimethylbenzaldehyde	33.1 ± 4.33	ND ^††^	28.4 ± 1.02	ND ^††^	ND ^††^	ND ^††^

^†^ 6.4 W power output, 1.5 mm air hole, VG based e-liquid containing 12 mg/mL nicotine, and 24 s puff interval were used; ^††^ ND indicates non-detected; ^†††^ <LOD indicates the measurement which is below the detection limit; ^††††^ <LOQ indicates the measurement which is below the quantification limit.

## Supplementary Materials

The following are available online at https://www.mdpi.com/1660-4601/17/16/5650/s1, Table S1. Summary of the study participants, Table S2. E-cigarette vaping patterns from the study subjects (*N* = 23), Figure S1. E-cigarette vaping topography observed from 23 e-cigarette users, Figure S2. Scheme of the carbonyl sampling system, Table S3. Retention times, calibration parameters, LODs, and LOQs for the selected carbonyls, Table S4. Impact of power outputs and base materials on carbonyl levels in e-vapor (mean ± standard deviation, *n* = 5), Table S5. Impact of flavoring agents on carbonyl levels in e-vapor (mean ± standard deviation, *n* = 5), Figure S3. Example of the top and bottom coil settings, Table S6. Carbonyls emitted from the e-cigarette and conventional cigarette. E-cigarette carbonyl levels were measured in this study and carbonyl emissions from cigarette were adopted from Fujioka and Shibamoto (2006), Table S7. Estimated daily carbonyl exposures for e-cigarette and cigarette users.
