# Supplementary material for: The Impact of Device Settings, Use Patterns, and Flavorings on Carbonyl Emissions from Electronic Cigarettes
Source: Int J Environ Res Public Health. 2020 Aug 5;17(16):5650. doi: 10.3390/ijerph17165650 (PMC7460324; doi:10.3390/ijerph17165650)
Supplement: Supplementary file 1 [file ijerph-17-05650-s001.pdf]

Supplementary material

1. Demographics of the study participants

The demographics of the study participants are summarized in Table S1.

Table S1. Summary of the study participants.

Number of subject	23
Age	25 ± 10 (18-52) years
Gender	21 men and 2 women
Ethnicity	16 White; 1 Black; 3 Asian; 6 others
Duration of E-cigarette use	1.4 ± 0.9 (0.4-4.0) years

2. E-cigarette use patterns of the 23 subjects in our study

Table S3 shows the mean, the standard deviation, and the range of e-cigarette vaping topography, device power output, and nicotine contents of the 23 subjects in our study. E-cigarette device power output ranged from 5-watt to 59.7-watt, with an average power output of 13.7-watt. The average nicotine content in e-liquids was 11.9 ± 10.0 mg/mL, with a maximum nicotine level of 36 mg/ml. Most subjects used VG-based e-liquids (14 out of 23 subjects), followed by PG:VG mixed e-liquid (7 subjects), and PG-based e-liquid (2 subjects).

Table S2. E-cigarette vaping patterns from the study subjects (N = 23).

**Parameters**	**Mean**	**Standard Deviation**	**Percentiles**						
			Min	10	25	50	75	90	Max
Puff volume (mL)	100.17	55.57	9.99	38.39	63.58	90.04	135.62	160.46	251.13
Puff duration (sec)	3.69	1.16	1.26	2.08	3.24	3.85	4.24	5.06	5.77
Puff interval (sec)	24.30	17.30	8.01	11.90	13.86	18.67	26.35	67.91	69.39
Power (W)	13.70	15.14	5.00	5.48	6.26	7.61	12.96	27.38	59.67
Nicotine (mg/mL)	11.92	10.04	0.00	3.00	3.00	12.00	19.50	24.00	36.00

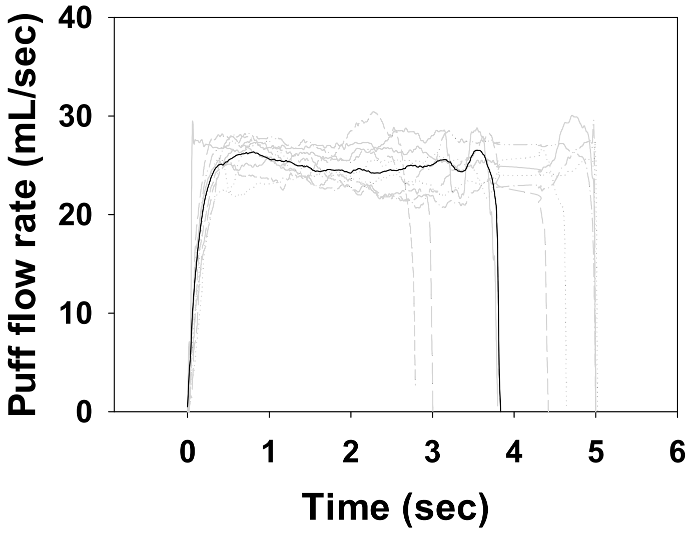

Figure S1. E-cigarette vaping topography observed from 23 e-cigarette users.

3. Chemical components of flavoring ingredients used in e-liquids (provided by the vendor/manufacturer)

The chemical components of the flavoring ingredients were only partially released by the vendors/manufactures. The strawberry (ripe), dragon fruit, menthol, and sweet cream flavors consist of natural/artificial flavors in propylene glycol (PG). The Bavarian cream flavor consists of natural/artificial flavors, PG, and water. The cinnamon flavor is composed of artificial flavors in ethyl alcohol. The bubblegum (fruity) flavor consists of natural/artificial flavors in PG and ethyl alcohol. The graham cracker flavor is composed of natural/artificial flavor in PG and water, with caramel color, corn syrup, ethyl alcohol, and salt.

4. The carbonyl sampling system

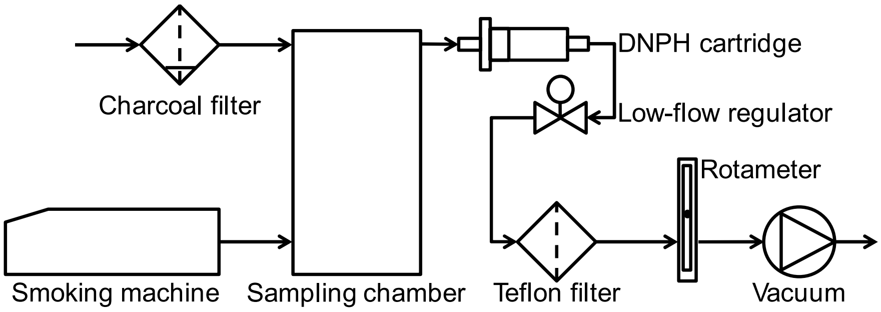

Figure S2. Scheme of the carbonyl sampling system

5. Carbonyl calibration table

Table S3. Retention times, calibration parameters, LODs, and LOQs for the selected carbonyls.

Chemical	Time (min)	Range (ng/μl)	Calibration parameters[†]		R^2	LOD (ng/30 puff)[††]	LOQ (ng/30 puff)[††]
			a	b			
Glyoxal	4.4	0.1–10.0	3.712×10^{-6}	0.1127	0.9982	49.6	165.4
Formaldehyde	4.9	0.1–20.0	3.417×10^{-6}	0.1052	0.9981	31.1	103.7
Acetaldehyde	7.3	0.1–10.0	4.796×10^{-6}	0.1351	0.9990	22.4	74.8
Diacetyl	8.3	0.1–10.0	1.654×10^{-6}	0.0981	0.9981	11.1	36.9
Acetone	10.8	0.1–10.0	6.131×10^{-6}	0.0931	0.9947	25.8	86.1
Vanillin	12.0	0.1–10.0	6.888×10^{-6}	0.2507	0.9967	4.58	15.3
Acrolein	11.5	0.1–10.0	5.276×10^{-6}	0.1753	0.9957	10.9	36.3
Propionaldehyde	12.8	0.1–10.0	4.655×10^{-6}	0.3606	0.9912	4.32	14.4
Acetylpropionyl	13.7	0.1–10.0	1.828×10^{-6}	0.2324	0.9944	8.61	28.7
Crotonaldehyde	15.8	0.1–10.0	6.869×10^{-6}	0.1955	0.9965	5.19	17.3
n-Butylaldehyde	17.7	0.1–10.0	6.744×10^{-6}	0.4268	0.9896	14.9	49.6
Benzaldehyde	20.1	0.1–10.0	1.002×10^{-5}	0.2561	0.9970	8.58	28.6
Isovaleraldehyde	22.0	0.1–10.0	1.045×10^{-5}	0.0960	0.9943	4.77	15.9
n-Valeraldehyde	22.8	0.1–10.0	7.352×10^{-6}	0.5103	0.9833	3.03	10.1
o-Tolualdehyde	24.3	0.1–10.0	1.778×10^{-5}	0.2090	0.9947	5.04	16.8
p-Tolualdehyde	24.9	0.1–10.0	4.480×10^{-6}	0.4780	0.9858	6.32	21.1
Cinnamaldehyde	25.7	0.1–10.0	1.642×10^{-5}	0.1297	0.9960	8.11	27.0
n-Hexaldehyde	28.0	0.1–10.0	1.591×10^{-5}	0.0620	0.9976	16.6	55.2
Dimethylbenzaldehyde	28.8	0.1–10.0	1.248×10^{-5}	0.1502	0.9829	0.36	1.19

[†] a and b indicate slope and intercept of the calibration equation, respectively;[††] LOD and LOQ were three- and ten-times standard deviation of 0.1 ng/μl sample (n = 7), respectively.

6. E-cigarette carbonyl emissions under different usepatterns

Table S4. Impact of power outputs and base materials on carbonyl levels in e-vapor (mean ± standard deviation, n = 5).

Carbonyl	Unit	Base material and power output (watts)†								
		VG			PG:VG (v:v=1:1)			PG		
		6.4W	14.7 W	31.3W	6.4W	14.7W	31.3W	6.4W	14.7W	31.3W
Glyoxal	ng/ puff	ND††	ND††	240.1 ± 13.7	ND††	ND††	ND††	ND††	ND††	ND††
Formaldehyde	μg/ puff	0.903 ± 0.0562	1.10 ± 0.0920	1.26 ± 0.127	0.927 ± 0.0474	1.15 ± 0.0653	1.96 ± 0.348	0.957 ± 0.0288	1.20 ± 0.0824	2.32 ± 0.0419
Acetaldehyde	μg/ puff	0.0917 ± 0.0181	0.0778 ± 0.044	0.0825 ± 0.0360	0.117 ± 0.0104	0.534 ± 0.0584	0.553 ± 0.0853	0.362 ± 0.0742	1.09 ± 0.0883	1.02 ± 0.0611
Acetone	ng/ puff	<LOD†††	ND††	<LOD†††	<LOD†††	ND††	ND††	<LOD†††	ND††	<LOD
Acrolein	ng/ puff	<LOQ††††	<LOQ††††	251.6 ± 51.9	42.6 ± 6.55	29.2 ± 7.81	199.2 ± 14.8	67.3 ± 14.8	97.5 ± 62.5	208.9 ± 89.6
Propionaldehyde	ng/ puff	ND††	ND††	ND††	ND††	ND††	ND††	ND††	ND††	24.0 ± 3.74
Crotonaldehyde	ng/ puff	29.8 ± 6.02	ND††	17.7 ± 0.08	ND††	ND††	33.6±4.4	ND††	ND††	54.0±12.3
n-Butylaldehyde	ng/ puff	ND††	ND††	156.1 ± 7.82	ND††	93.1 ± 28.1	402.1 ± 16.9	25.5 ± 2.2	28.4 ± 2.9	422.9 ± 9.34
Benzaldehyde	ng/ puff	23.1 ± 12.4	ND††	27.7 ± 1.75	ND††	ND††	31.2 ± 2.69	ND††	ND††	31.3 ± 2.82
Isovaleraldehyde	ng/ puff	ND††	ND††	68.1 ± 13.5	ND††	ND††	136.9 ± 8.21	ND††	ND††	86.4 ± 44.3
n-Valeraldehyde	ng/ puff	81.1 ± 19.4	ND††	70.7 ± 20.0	ND††	53.4 ± 13.3	ND††	ND††	ND††	ND††
o-Tolualdehyde	ng/ puff	ND††	ND††	ND††	ND††	ND††	198.0 ± 15.0	42.0 ± 3.80	ND††	329.1 ± 68.4
p-Tolualdehyde	ng/ puff	18.1 ± 1.63	ND††	ND††	ND††	ND††	ND††	ND††	ND††	ND††

n-Hexaldehyde	ng/ puff	248.1 ± 65.1	563.1 ± 142.	ND[††]	ND[††]	54.0 ± 4.49	ND[††]	ND[††]	130.4 ± 34.8	ND[††]
Dimethylbenzaldehyde	ng/ puff	ND[††]	ND[††]	ND[††]	ND[††]	ND[††]	31.4 ± 4.03	ND[††]	ND[††]	35.8 ± 3.77

[†] 1.5 mm air hole, 12 mg/ml nicotine, and 90 ml puff volume, 3.8 sec puff duration and 24 sec puff interval were used; [††] ND indicates non-detected; [†††] <LOD indicates the measurement which is below the detection limit; [††††] <LOQ indicates the measurement which is below the quantification limit.

Table S5. Impact of flavoring agents on carbonyl levels in e-vapor (mean ± standard deviation, n = 5).

Carbonyl	Unit	Flavoring agents (10% by volume, 1% for cinnamon flavor in VG-base) [†]							
		Strawberry	Dragon Fruit	Menthol	Cinnamon	Bavarian cream	Sweet cream	Bubble gum	Graham cracker
Glyoxal	ng/puff	ND[††]	ND[††]	ND[††]	ND[††]	ND[††]	ND[††]	ND[††]	ND[††]
Formaldehyde	µg/puff	1.26 ± 0.116	1.18 ± 0.035	0.951 ± 0.0501	0.672 ± 0.195	0.624 ± 0.0164	0.607 ± 0.0421	0.703 ± 0.0238	0.486 ± 0.0711
Acetaldehyde	ng/puff	49.0 ± 21.4	30.5 ± 1.24	30.4 ± 1.96	<LOQ[††††]	<LOQ[††††]	<LOQ[††††]	30.2 ± 2.35	<LOQ[††††]
Diacetyl	ng/puff	ND[††]	ND[††]	ND[††]	ND[††]	21.1 ± 11.7	86.4 ± 2.89	ND[††]	34.9 ± 16.8
Acetone	ng/puff	<LOD[†††]	<LOD[†††]	<LOQ[††††]	<LOD[†††]	ND[††]	ND[††]	ND[††]	<LOD[†††]
Acrolein	ng/puff	28.4 ± 8.92	20.9 ± 5.99	20.3 ± 1.81	29.0 ± 5.55	ND[††]	ND[††]	19.5 ± 4.18	131.2 ± 21.9
Vanillin	ng/puff	ND[††]	ND[††]	ND[††]	ND[††]	177.4 ± 60.1	178.5 ± 65.8	45.2 ± 3.15	184.4 ± 27.0
Propionaldehyde	ng/puff	ND[††]	ND[††]	ND[††]	ND[††]	ND[††]	ND[††]	ND[††]	ND[††]
Acetylpropionyl	ng/puff	ND[††]	ND[††]	ND[††]	ND[††]	ND[††]	ND[††]	ND[††]	ND[††]
Crotonaldehyde	ng/puff	32.5 ± 1.65	ND[††]	ND[††]	29.8 ± 3.86	ND[††]	19.0 ± 0.41	ND[††]	ND[††]
n-Butylaldehyde	ng/puff	ND[††]	29.4 ± 4.71	28.9 ± 4.01	ND[††]	ND[††]	ND[††]	27.3 ± 4.81	ND[††]
Benzaldehyde	ng/puff	29.2 ± 2.95	31.3 ± 5.48	30.4 ± 5.41	27.8 ± 2.47	26.8 ± 0.58	ND[††]	27.6 ± 2.55	25.0 ± 2.71
Isovaleraldehyde	ng/puff	16.8 ± 1.57	ND[††]	ND[††]	17.3 ± 0.85	33.6 ± 3.73	24.4 ± 6.12	ND[††]	ND[††]
n-Valeraldehyde	ng/puff	24.1 ± 3.65	ND[††]	ND[††]	25.3 ± 6.08	19.7±1.91	17.2 ± 0.14	18.9 ± 1.55	ND[††]
o-Tolualdehyde	ng/puff	ND[††]	29.3 ± 5.37	32.1 ± 4.65	26.1 ± 7.87	ND[††]	60.5 ± 2.34	62.3 ± 13.6	ND[††]

p-Tolualdehyde	ng/ puff	18.9 ± 1.74	ND††	17.7 ± 0.37	ND††	74.2 ± 9.65	51.4 ± 3.22	ND††	ND††
Cinnamaldehyde	ng/ puff	ND††	ND††	ND††	473.1 ± 234.	ND††	ND††	ND††	ND††
n-Hexaldehyde	ng/ puff	205.6 ± 7.54	179.0 ± 36.9	139.4 ± 15.5	160.4 ± 35.9	ND††	ND††	154.8 ± 3.28	ND††
Dimethylbenzaldehyde	ng/ puff	ND††	ND††	ND††	ND††	ND††	ND††	ND††	ND††

† 6.4W power output, 1.5 mm air hole, 90 ml puff volume, 3.8 sec puff duration, and 24 sec puff interval were used; †† ND indicates non-detected; ††† <LOD indicates the measurement which is below the detection limit; †††† <LOQ indicates the measurement which is below the quantification limit.

7. E-cigarette coil setting

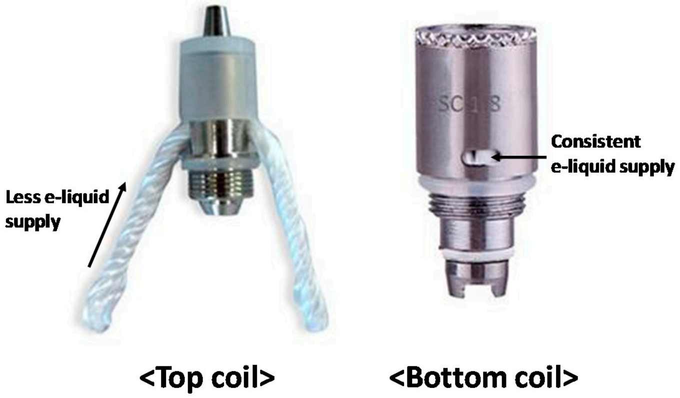

Figure S3. Example of the top and bottom coil settings (obtained from https://www.smokshop.com/blogs/news/15508169-heads-or-tails-bottom-and-top-coils).

8. Exposure Estimation

Carbonyl exposure distributions were estimated using the measured e-cigarette and reported cigarette carbonyl emission data (Table S6). Daily carbonyl exposures (weighted average) were estimated based on the reported e-cigarette and cigarette use patterns (Dautzenberg and Bricard, 2015; Jamal et al., 2018) (Table S7). 50% of e-cigarette users vaped 55-236 puffs/day, 30% of them puffed 236-346 puffs/day, and 19% of users puffed 346-600 puffs/day (Dautzenberg and Bricard, 2015). Based on the 2016 National Health Interview Survey (NHIS), 25%, 39%, 28.4%, and 7.5% of conventional cigarette users smoked 1-9, 10-19, 20-29, and more than 30 cigarettes/day respectively (Jamal et al., 2018). Estimated average daily carbonyl exposure values and corresponding standard deviations were used to generate exposure distributions using the Monte Carlo method (Figure 4 in main text, lognormal distribution, n=10,000).

Table S6. Carbonyls emitted from the e-cigarette and conventional cigarette. E-cigarette carbonyl levels were measured in this study and carbonyl emissions from cigarette were adopted from Fujioka and Shibamoto (2006).

Category	Unit	Formaldehyde	Acetaldehyde	Acrolein	Glyoxal	Diacetyl
E-cigarette	ng/puff	941.9 ± 254.0	54.0 ± 43.5	54.5 ± 88.2	43.6 ± 97.1	8.37 ± 22.3
Cigarette	μg/pack	145.5 ± 7.38	1756.6 ± 37.3	364.8 ± 16.8	3.16 ± 0.16	335.9 ± 16.5

Table S7. Estimated daily carbonyl exposures for e-cigarette and cigarette users.

Category	Unit	Formaldehyde	Acetaldehyde	Acrolein	Glyoxal	Diacetyl
E-cigarette	μg/day	201.0 ± 32.2	11.9 ± 1.67	12.0 ± 1.68	9.60 ± 1.48	1.87 ± 1.06
Cigarette	μg/day	101.7 ± 18.0	1224.4 ± 810.2	254.7 ± 60.7	2.22 ± 1.18	234.6 ± 53.9

References

Dautzenberg, B.; Bricard, D. Real-Time Characterization of E-Cigarettes Use: The 1 Million Puffs Study. *J. Addict. Res. Ther.* **2015**, *6*, 1–5.

Fujioka, K.; Shibamoto, T. Determination of toxic carbonyl compounds in cigarette smoke. *Environ. Toxicol* **2006**, *21*, 47–54.
Jamal, A.; Phillips, E.; Gentzke, A.S.; Homa, D.M.; Babb, S.D.; King, B.A.; Neff, L.J. Current cigarette smoking among adults—United States, 2016. *Morbidity and Mortality Weekly Report* **2018**, *67*, 53.
